# [*N*,*N*,*N*′,*N*′-Tetra­kis(benzimidazol-2-yl­meth­yl)cyclo­hexane-1,2-diamine]­nickel(II) dinitrate dihydrate

**DOI:** 10.1107/S1600536808031553

**Published:** 2008-10-04

**Authors:** Dan Zhan, Zuo-An Xiao

**Affiliations:** a Department of Chemical and Biological Science, Xiangfan University, Xiangfan 441053, People’s Republic of China

## Abstract

In the title compound, [Ni(C_38_H_38_N_10_)](NO_3_)_2_·2H_2_O, the Ni^II^ ion is located on a crystallographic twofold rotation axis and is in a distorted octa­hedral coordination environment. The crystal structure is stablized by inter­molecular N—H⋯O and C—H⋯O hydrogen bonds, and weak C—H⋯π inter­actions. The O atoms of the unique nitrate ion are disordered over two sites with occupancies of 0.63 (1) and 0.37 (1). In addition, the O atom of the unique solvent water mol­ecule is disorded over two sites with equal occupancies.

## Related literature

For background information, see: Oki *et al.* (1996 ▶); Hendriks *et al.* (1982 ▶); Main (1992 ▶); Zhao *et al.* (2005 ▶). For the structure of the free ligand of the title compound, see: Li *et al.* (2005 ▶).
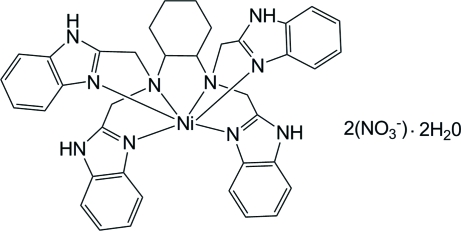


## Experimental

### 

#### Crystal data

[Ni(C_38_H_38_N_10_)](NO_3_)_2_·2H_2_O
*M* *_r_* = 853.55
Monoclinic, 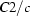

*a* = 15.3395 (16) Å
*b* = 13.1695 (14) Å
*c* = 19.606 (2) Å
β = 98.501 (2)°
*V* = 3917.2 (7) Å^3^
*Z* = 4
Mo *K*α radiation
μ = 0.56 mm^−1^
*T* = 292 (2) K
0.32 × 0.20 × 0.10 mm

#### Data collection

Bruker SMART APEX CCD diffractometer
Absorption correction: multi-scan (*SADABS*; Sheldrick, 1996 ▶) *T*_min_ = 0.840, *T*_max_ = 0.946
10917 measured reflections
3847 independent reflections
2618 reflections with *I* > 2σ(*I*)
*R*_int_ = 0.074

#### Refinement

*R*[*F* ^2^ > 2σ(*F* ^2^)] = 0.056
*wR*(*F* ^2^) = 0.133
*S* = 0.95
3847 reflections
303 parameters
65 restraints
H atoms treated by a mixture of independent and constrained refinement
Δρ_max_ = 0.43 e Å^−3^
Δρ_min_ = −0.34 e Å^−3^

### 

Data collection: *SMART* (Bruker, 2007 ▶); cell refinement: *SAINT-Plus* (Bruker, 2007 ▶); data reduction: *SAINT-Plus*; program(s) used to solve structure: *SHELXS97* (Sheldrick, 2008 ▶); program(s) used to refine structure: *SHELXL97* (Sheldrick, 2008 ▶); molecular graphics: *PLATON* (Spek, 2003 ▶); software used to prepare material for publication: *SHELXTL* (Sheldrick, 2008 ▶).

## Supplementary Material

Crystal structure: contains datablocks global, I. DOI: 10.1107/S1600536808031553/lh2699sup1.cif

Structure factors: contains datablocks I. DOI: 10.1107/S1600536808031553/lh2699Isup2.hkl

Additional supplementary materials:  crystallographic information; 3D view; checkCIF report

## Figures and Tables

**Table:** 

Ni1—N2	2.061 (2)
Ni1—N4	2.071 (3)
Ni1—N1	2.190 (3)

**Table:** 

N2—Ni1—N2^i^	175.20 (15)
N2—Ni1—N4	90.83 (10)
N2—Ni1—N4^i^	91.53 (10)
N2—Ni1—N1^i^	81.44 (10)
N4—Ni1—N1^i^	79.11 (10)
N2—Ni1—N1	94.90 (10)
N4—Ni1—N1	158.69 (10)
N1^i^—Ni1—N1	81.47 (14)

Symmetry code: (i) 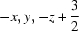.

**Table 2:** Hydrogen-bond geometry (Å, °) *Cg*1 and *Cg*2 are the centroids defined by atoms C7–C12 and C14–C19, respectively.

*D*—H⋯*A*	*D*—H	H⋯*A*	*D*⋯*A*	*D*—H⋯*A*
N3—H3⋯O1^ii^	0.86	1.94	2.774 (10)	165
N5—H5⋯O2^iii^	0.86	2.14	2.923 (7)	151
C4—H4*B*⋯O4	0.97	2.37	3.268 (12)	154
C11—H11⋯*Cg*1^iv^	0.93	2.73	3.542	147
C16—H16⋯*Cg*2^v^	0.93	2.79	3.680	160

Symmetry codes: (ii) 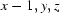; (iii) 
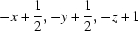
; (iv) 
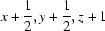
; (v) 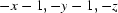.

**Fig. 1.**
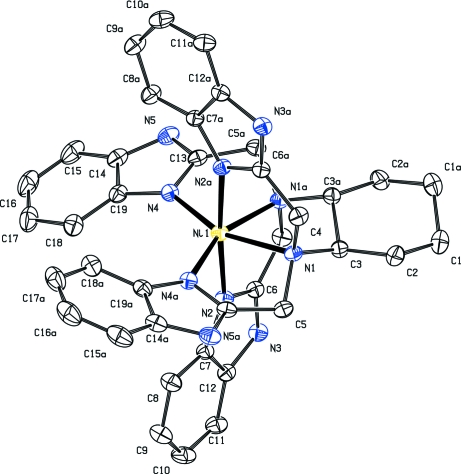
The molecular structure of (I), showing the atom-numbering scheme. Displacement ellipsoids are drawn at the 30% probability level. The nitrate anions, water molecules and H atoms are omitted for clarity [symmetry code: (a) -x, y, -z+3/2].

**Fig. 2.**
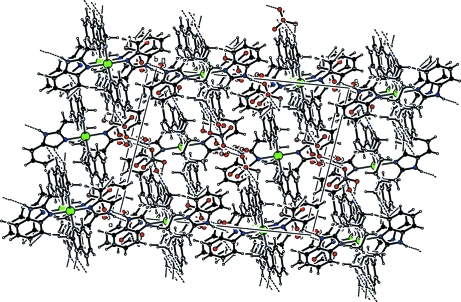
Part of the crystal structure showing the linking of molecules by H-bonding and weak C—H···π interactions as dashed lines.

## supplementary crystallographic information

### Comment

*N*,*N*,*N*',*N*'-Tetrakis(2-benzimidazolymethyl) cyclohexane-1,2-diamine (CTB) is a polybenzimidazole ligand, which has the advantage that the basicity of the coordinating group approximates that of histidine (pKb: histidine = 7.96 and benzimidazole = 8.47; Main, 1992). Recently, studies of ligand CTB and its metal coordination compounds have been widely carried out (Li *et al.*,2005; Zhao *et al.*, 2005). In a continuation of this work, the title compound, (I), was prepared as part of a series of syntheses to produce new benzimidazole derivatives. We report the crystal stucture of the title compound herein.

In the molecule structure of (I), the Ni^II^ ion is located on a crystallographic twofold rotation axis and is is coordinated by four benzimidazolyl(bzim) N atoms and two amino N atoms of the ligand CTB, in a distorted octahedral environment (Fig.1). The amino N atoms are slightly further away from the Ni^II^ ion than the benzimidazolyl N atoms. The Ni-N bond lengths are similar to the values reported in a related structure (Oki *et al.*,1996). As shown in Fig. 2, the crystal structure is stablized by intermolecular N—H···O, C—H···O hydrogen bonds and weak C—H···π interactions.

### Experimental

All reagents and solvents were used as obtained without further purification. The ligand CTB was prepared according to literature methods (Hendriks *et al.*, 1982). Compound (I) was synthesized by refluxing stoichiometric quantities (1:1 molar ratio) of CTB (0.64 g, 1 mmol) and nickel(II) dinitrate hexahydrate (0.29 g, 1 mmol) in 95% ethanol (30 ml) at 333 K for 6 h. The solution was cooled to room temperature, filtered and evaporated to obtain the product (yield 72%). Crystals of (I) were grown from an ethanol solution by slow evaporation.

### Refinement

In (I), the nitrate O atoms are disordered over two positions with the final refined occupancies of 0.63 (1):0.37 (1). Water atom O4 is also disordered over two positions with both the occupancies being set to 0.5. H atoms bonded to water molecules were not located and were not included in the refinement but are included in the molecular formula. All H atoms (except for H3A) were included in geometrical positions with C—H=0.97 Å (methylene), 0.93Å (aromatic), 0.86Å (imine) and all the *U*_iso_values were set 1.2 times of their carrier atoms. The positional parameters of atom H3A were refined.

### Crystal data

**Table:**

[Ni(C_38_H_38_N_10_)](NO_3_)_2_·2H_2_O	*F*(000) = 1784
*M**_r_* = 853.55	*D*_x_ = 1.447 Mg m^−^^3^
Monoclinic, *C*2/*c*	Mo *K*α radiation, λ = 0.71073 Å
Hall symbol: -C 2yc	Cell parameters from 2061 reflections
*a* = 15.3395 (16) Å	θ = 2.4–22.1°
*b* = 13.1695 (14) Å	µ = 0.56 mm^−^^1^
*c* = 19.606 (2) Å	*T* = 292 K
β = 98.501 (2)°	Plate, purple
*V* = 3917.2 (7) Å^3^	0.32 × 0.20 × 0.10 mm
*Z* = 4	

### Data collection

**Table:**

Bruker SMART APEX CCD diffractometer	3847 independent reflections
Radiation source: fine-focus sealed tube	2618 reflections with *I* > 2σ(*I*)
graphite	*R*_int_ = 0.074
φ and ω scans	θ_max_ = 26.0°, θ_min_ = 2.1°
Absorption correction: multi-scan (SADABS; Sheldrick, 1996)	*h* = −18→17
*T*_min_ = 0.840, *T*_max_ = 0.946	*k* = −10→16
10917 measured reflections	*l* = −24→24

### Refinement

**Table:**

Refinement on *F*^2^	Primary atom site location: structure-invariant direct methods
Least-squares matrix: full	Secondary atom site location: difference Fourier map
*R*[*F*^2^ > 2σ(*F*^2^)] = 0.056	Hydrogen site location: inferred from neighbouring sites
*wR*(*F*^2^) = 0.133	H atoms treated by a mixture of independent and constrained refinement
*S* = 0.95	*w* = 1/[σ^2^(*F*_o_^2^) + (0.0618*P*)^2^] where *P* = (*F*_o_^2^ + 2*F*_c_^2^)/3
3847 reflections	(Δ/σ)_max_ = 0.001
303 parameters	Δρ_max_ = 0.43 e Å^−^^3^
65 restraints	Δρ_min_ = −0.34 e Å^−^^3^

### Special details

**Table:**

Geometry. All e.s.d.'s (except the e.s.d. in the dihedral angle between two l.s. planes) are estimated using the full covariance matrix. The cell e.s.d.'s are taken into account individually in the estimation of e.s.d.'s in distances, angles and torsion angles; correlations between e.s.d.'s in cell parameters are only used when they are defined by crystal symmetry. An approximate (isotropic) treatment of cell e.s.d.'s is used for estimating e.s.d.'s involving l.s. planes.
Refinement. Refinement of *F*^2^ against ALL reflections. The weighted *R*-factor *wR* and goodness of fit *S* are based on *F*^2^, conventional *R*-factors *R* are based on *F*, with *F* set to zero for negative *F*^2^. The threshold expression of *F*^2^ > σ(*F*^2^) is used only for calculating *R*-factors(gt) *etc*. and is not relevant to the choice of reflections for refinement. *R*-factors based on *F*^2^ are statistically about twice as large as those based on *F*, and *R*- factors based on ALL data will be even larger.

### Fractional atomic coordinates and isotropic or equivalent isotropic displacement parameters (Å^2^)

**Table:**

	*x*	*y*	*z*	*U*_iso_*/*U*_eq_	Occ. (<1)
Ni1	0.0000	0.06922 (5)	0.7500	0.0383 (2)	
N1	0.08499 (16)	0.1952 (2)	0.79130 (13)	0.0411 (7)	
N2	0.03564 (16)	0.0758 (2)	0.65276 (12)	0.0420 (7)	
N3	0.01367 (17)	0.1461 (2)	0.54912 (12)	0.0461 (7)	
H3	−0.0083	0.1852	0.5158	0.055*	
N4	−0.11459 (17)	−0.0078 (2)	0.71196 (12)	0.0419 (7)	
N5	−0.25584 (18)	0.0113 (3)	0.66910 (14)	0.0552 (8)	
H5	−0.3063	0.0401	0.6586	0.066*	
N6	0.8994 (3)	0.3077 (5)	0.4093 (3)	0.139 (2)	
O1	0.9736 (6)	0.2879 (13)	0.4445 (5)	0.268 (8)	0.630 (9)
O2	0.8880 (3)	0.3273 (6)	0.3471 (3)	0.119 (3)	0.630 (9)
O3	0.8392 (5)	0.3165 (5)	0.4456 (4)	0.142 (3)	0.630 (9)
O1'	0.9621 (5)	0.2465 (7)	0.4281 (4)	0.058 (3)	0.370 (9)
O2'	0.9055 (8)	0.4029 (8)	0.4173 (8)	0.161 (7)	0.370 (9)
O3'	0.8270 (19)	0.2712 (14)	0.377 (3)	0.29 (12)*	0.370 (9)
O4	0.0215 (8)	0.4055 (10)	0.9433 (5)	0.169 (6)	0.50 (2)
O4'	0.0237 (12)	0.491 (2)	0.9646 (10)	0.279 (12)	0.50 (2)
C1	0.0479 (3)	0.4797 (3)	0.7444 (2)	0.0738 (12)	
H1A	0.0765	0.5412	0.7635	0.089*	
H1B	0.0512	0.4784	0.6954	0.089*	
C2	0.0954 (2)	0.3874 (3)	0.7790 (2)	0.0609 (10)	
H2A	0.1560	0.3873	0.7704	0.073*	
H2B	0.0958	0.3916	0.8284	0.073*	
C3	0.0508 (2)	0.2889 (3)	0.75205 (18)	0.0454 (8)	
H3A	0.063 (2)	0.280 (2)	0.7048 (16)	0.054*	
C4	0.0837 (2)	0.2029 (3)	0.86709 (16)	0.0494 (9)	
H4A	0.1394	0.1790	0.8917	0.059*	
H4B	0.0767	0.2734	0.8795	0.059*	
C5	0.1735 (2)	0.1638 (3)	0.77788 (18)	0.0492 (9)	
H5A	0.2189	0.2039	0.8052	0.059*	
H5B	0.1787	0.1722	0.7295	0.059*	
C6	−0.0106 (2)	0.1420 (3)	0.61190 (15)	0.0412 (8)	
C7	0.0944 (2)	0.0319 (3)	0.61274 (15)	0.0412 (8)	
C8	0.1581 (2)	−0.0419 (3)	0.62782 (19)	0.0570 (10)	
H8	0.1678	−0.0735	0.6707	0.068*	
C9	0.2069 (3)	−0.0668 (3)	0.5766 (2)	0.0644 (11)	
H9	0.2502	−0.1165	0.5852	0.077*	
C10	0.1936 (3)	−0.0204 (3)	0.51282 (19)	0.0658 (11)	
H10	0.2289	−0.0386	0.4801	0.079*	
C11	0.1299 (2)	0.0515 (3)	0.49675 (17)	0.0561 (10)	
H11	0.1204	0.0823	0.4536	0.067*	
C12	0.0804 (2)	0.0764 (3)	0.54747 (15)	0.0441 (8)	
C13	−0.1819 (2)	0.0548 (3)	0.70208 (16)	0.0453 (8)	
C14	−0.2356 (2)	−0.0880 (3)	0.65533 (18)	0.0546 (10)	
C15	−0.2841 (3)	−0.1652 (4)	0.6196 (2)	0.0793 (14)	
H15	−0.3432	−0.1566	0.6015	0.095*	
C16	−0.2408 (3)	−0.2551 (4)	0.6122 (2)	0.0862 (15)	
H16	−0.2710	−0.3084	0.5883	0.103*	
C17	−0.1518 (3)	−0.2677 (3)	0.6401 (2)	0.0800 (14)	
H17	−0.1248	−0.3298	0.6346	0.096*	
C18	−0.1031 (3)	−0.1913 (3)	0.67524 (18)	0.0601 (10)	
H18	−0.0439	−0.2002	0.6931	0.072*	
C19	−0.1463 (2)	−0.1002 (3)	0.68280 (16)	0.0468 (9)	

### Atomic displacement parameters (Å^2^)

**Table:**

	*U*^11^	*U*^22^	*U*^33^	*U*^12^	*U*^13^	*U*^23^
Ni1	0.0387 (4)	0.0453 (4)	0.0307 (3)	0.000	0.0044 (2)	0.000
N1	0.0380 (15)	0.0490 (17)	0.0366 (14)	−0.0011 (13)	0.0062 (11)	0.0024 (13)
N2	0.0414 (15)	0.0507 (17)	0.0342 (14)	0.0041 (14)	0.0066 (11)	0.0015 (14)
N3	0.0536 (17)	0.0550 (18)	0.0292 (14)	0.0030 (15)	0.0044 (12)	0.0085 (13)
N4	0.0433 (16)	0.0469 (17)	0.0347 (15)	−0.0039 (14)	0.0028 (12)	−0.0051 (13)
N5	0.0393 (17)	0.071 (2)	0.0544 (19)	−0.0096 (16)	0.0027 (14)	0.0030 (17)
N6	0.087 (4)	0.119 (5)	0.203 (8)	0.000 (4)	−0.007 (5)	0.038 (5)
O1	0.179 (10)	0.317 (15)	0.290 (14)	0.021 (10)	−0.025 (9)	0.206 (12)
O2	0.079 (4)	0.176 (7)	0.097 (4)	0.031 (4)	−0.003 (3)	0.091 (5)
O3	0.127 (6)	0.125 (6)	0.191 (7)	0.023 (5)	0.082 (5)	−0.025 (5)
O1'	0.052 (5)	0.076 (6)	0.044 (4)	0.027 (4)	0.005 (3)	0.023 (4)
O2'	0.115 (9)	0.170 (13)	0.184 (13)	0.027 (9)	−0.025 (8)	0.000 (10)
O4	0.239 (11)	0.123 (10)	0.149 (8)	0.023 (7)	0.043 (7)	−0.076 (6)
O4'	0.311 (17)	0.28 (2)	0.247 (17)	0.064 (15)	0.042 (12)	0.007 (16)
C1	0.084 (3)	0.050 (2)	0.088 (3)	−0.011 (2)	0.013 (3)	−0.001 (2)
C2	0.061 (2)	0.053 (2)	0.069 (3)	−0.011 (2)	0.012 (2)	−0.003 (2)
C3	0.0481 (19)	0.045 (2)	0.0440 (19)	−0.0006 (17)	0.0101 (16)	0.0002 (17)
C4	0.047 (2)	0.059 (2)	0.0403 (19)	−0.0071 (18)	0.0007 (15)	−0.0039 (17)
C5	0.0388 (19)	0.058 (2)	0.050 (2)	−0.0037 (17)	0.0064 (15)	0.0022 (18)
C6	0.0418 (18)	0.047 (2)	0.0339 (17)	−0.0018 (16)	0.0043 (14)	−0.0007 (16)
C7	0.0434 (18)	0.047 (2)	0.0335 (17)	−0.0016 (16)	0.0075 (14)	−0.0021 (15)
C8	0.060 (2)	0.063 (3)	0.049 (2)	0.013 (2)	0.0106 (18)	0.0008 (19)
C9	0.063 (2)	0.073 (3)	0.059 (2)	0.017 (2)	0.0133 (19)	−0.008 (2)
C10	0.060 (3)	0.094 (3)	0.047 (2)	−0.002 (2)	0.0188 (19)	−0.017 (2)
C11	0.059 (2)	0.075 (3)	0.0358 (18)	−0.004 (2)	0.0118 (17)	−0.0019 (19)
C12	0.0461 (19)	0.051 (2)	0.0345 (17)	−0.0066 (18)	0.0051 (14)	−0.0027 (16)
C13	0.0402 (19)	0.059 (2)	0.0372 (18)	−0.0065 (18)	0.0069 (14)	0.0005 (17)
C14	0.052 (2)	0.068 (3)	0.044 (2)	−0.016 (2)	0.0081 (17)	0.0011 (19)
C15	0.073 (3)	0.094 (4)	0.069 (3)	−0.042 (3)	0.005 (2)	−0.005 (3)
C16	0.105 (4)	0.079 (4)	0.073 (3)	−0.048 (3)	0.010 (3)	−0.014 (3)
C17	0.121 (4)	0.055 (3)	0.067 (3)	−0.024 (3)	0.023 (3)	−0.009 (2)
C18	0.076 (3)	0.058 (3)	0.046 (2)	−0.008 (2)	0.0063 (19)	−0.0029 (19)
C19	0.057 (2)	0.053 (2)	0.0319 (17)	−0.0085 (18)	0.0091 (15)	0.0018 (16)

### Geometric parameters (Å, °)

**Table:**

Ni1—N2	2.061 (2)	C2—H2B	0.9700
Ni1—N2^i^	2.062 (2)	C3—C3^i^	1.549 (6)
Ni1—N4	2.071 (3)	C3—H3A	0.98 (3)
Ni1—N4^i^	2.071 (3)	C4—C6^i^	1.485 (4)
Ni1—N1^i^	2.190 (3)	C4—H4A	0.9700
Ni1—N1	2.190 (3)	C4—H4B	0.9700
N1—C5	1.479 (4)	C5—C13^i^	1.489 (5)
N1—C4	1.493 (4)	C5—H5A	0.9700
N1—C3	1.506 (4)	C5—H5B	0.9700
N2—C6	1.318 (4)	C6—C4^i^	1.485 (4)
N2—C7	1.404 (4)	C7—C8	1.379 (5)
N3—C6	1.339 (4)	C7—C12	1.395 (4)
N3—C12	1.378 (4)	C8—C9	1.378 (5)
N3—H3	0.8600	C8—H8	0.9300
N4—C13	1.313 (4)	C9—C10	1.378 (5)
N4—C19	1.400 (4)	C9—H9	0.9300
N5—C13	1.348 (4)	C10—C11	1.364 (5)
N5—C14	1.379 (5)	C10—H10	0.9300
N5—H5	0.8600	C11—C12	1.378 (4)
N6—O2	1.233 (6)	C11—H11	0.9300
N6—O3	1.252 (6)	C13—C5^i^	1.489 (5)
N6—O2'	1.265 (8)	C14—C15	1.387 (5)
N6—O1'	1.267 (7)	C14—C19	1.405 (5)
N6—O1	1.268 (7)	C15—C16	1.375 (7)
N6—O3'	1.286 (9)	C15—H15	0.9300
O4'—O4'^ii^	1.68 (4)	C16—C17	1.403 (6)
C1—C1^i^	1.519 (8)	C16—H16	0.9300
C1—C2	1.524 (5)	C17—C18	1.376 (5)
C1—H1A	0.9700	C17—H17	0.9300
C1—H1B	0.9700	C18—C19	1.389 (5)
C2—C3	1.524 (5)	C18—H18	0.9300
C2—H2A	0.9700		
			
N2—Ni1—N2^i^	175.20 (15)	C2—C3—C3^i^	114.5 (2)
N2—Ni1—N4	90.83 (10)	N1—C3—H3A	106.6 (19)
N2^i^—Ni1—N4	91.53 (10)	C2—C3—H3A	106.6 (19)
N2—Ni1—N4^i^	91.53 (10)	C3^i^—C3—H3A	106.6 (19)
N2^i^—Ni1—N4^i^	90.82 (10)	C6^i^—C4—N1	111.2 (3)
N4—Ni1—N4^i^	121.31 (16)	C6^i^—C4—H4A	109.4
N2—Ni1—N1^i^	81.44 (10)	N1—C4—H4A	109.4
N2^i^—Ni1—N1^i^	94.90 (10)	C6^i^—C4—H4B	109.4
N4—Ni1—N1^i^	79.11 (10)	N1—C4—H4B	109.4
N4^i^—Ni1—N1^i^	158.69 (11)	H4A—C4—H4B	108.0
N2—Ni1—N1	94.90 (10)	N1—C5—C13^i^	105.6 (3)
N2^i^—Ni1—N1	81.43 (10)	N1—C5—H5A	110.6
N4—Ni1—N1	158.69 (10)	C13^i^—C5—H5A	110.6
N4^i^—Ni1—N1	79.11 (10)	N1—C5—H5B	110.6
N1^i^—Ni1—N1	81.47 (14)	C13^i^—C5—H5B	110.6
C5—N1—C4	110.1 (2)	H5A—C5—H5B	108.8
C5—N1—C3	113.4 (2)	N2—C6—N3	112.8 (3)
C4—N1—C3	113.5 (3)	N2—C6—C4^i^	123.3 (3)
C5—N1—Ni1	103.5 (2)	N3—C6—C4^i^	123.8 (3)
C4—N1—Ni1	108.93 (19)	C8—C7—C12	120.1 (3)
C3—N1—Ni1	106.76 (18)	C8—C7—N2	131.6 (3)
C6—N2—C7	105.4 (3)	C12—C7—N2	108.3 (3)
C6—N2—Ni1	113.4 (2)	C9—C8—C7	117.1 (3)
C7—N2—Ni1	141.2 (2)	C9—C8—H8	121.5
C6—N3—C12	107.6 (3)	C7—C8—H8	121.5
C6—N3—H3	126.2	C8—C9—C10	122.2 (4)
C12—N3—H3	126.2	C8—C9—H9	118.9
C13—N4—C19	105.5 (3)	C10—C9—H9	118.9
C13—N4—Ni1	110.6 (2)	C11—C10—C9	121.4 (3)
C19—N4—Ni1	142.9 (2)	C11—C10—H10	119.3
C13—N5—C14	107.4 (3)	C9—C10—H10	119.3
C13—N5—H5	126.3	C10—C11—C12	116.8 (3)
C14—N5—H5	126.3	C10—C11—H11	121.6
O2—N6—O3	122.3 (5)	C12—C11—H11	121.6
O2—N6—O2'	85.0 (9)	C11—C12—N3	131.8 (3)
O3—N6—O2'	83.4 (9)	C11—C12—C7	122.4 (3)
O2—N6—O1'	114.3 (7)	N3—C12—C7	105.8 (3)
O3—N6—O1'	119.1 (7)	N4—C13—N5	113.0 (3)
O2'—N6—O1'	123.7 (6)	N4—C13—C5^i^	122.1 (3)
O2—N6—O1	124.5 (6)	N5—C13—C5^i^	124.8 (3)
O3—N6—O1	112.8 (6)	N5—C14—C15	132.4 (4)
O2'—N6—O1	95.3 (11)	N5—C14—C19	105.5 (3)
O2'—N6—O3'	118.3 (7)	C15—C14—C19	122.0 (4)
O1'—N6—O3'	117.9 (7)	C16—C15—C14	116.9 (4)
O1—N6—O3'	146.2 (13)	C16—C15—H15	121.5
C1^i^—C1—C2	110.1 (3)	C14—C15—H15	121.5
C1^i^—C1—H1A	109.6	C15—C16—C17	121.2 (5)
C2—C1—H1A	109.6	C15—C16—H16	119.4
C1^i^—C1—H1B	109.6	C17—C16—H16	119.4
C2—C1—H1B	109.6	C18—C17—C16	122.3 (5)
H1A—C1—H1B	108.1	C18—C17—H17	118.9
C3—C2—C1	111.4 (3)	C16—C17—H17	118.9
C3—C2—H2A	109.4	C17—C18—C19	116.9 (4)
C1—C2—H2A	109.4	C17—C18—H18	121.6
C3—C2—H2B	109.4	C19—C18—H18	121.6
C1—C2—H2B	109.4	C18—C19—N4	130.6 (3)
H2A—C2—H2B	108.0	C18—C19—C14	120.8 (4)
N1—C3—C2	114.5 (3)	N4—C19—C14	108.5 (3)
N1—C3—C3^i^	107.3 (2)		
			
N2—Ni1—N1—C5	−55.5 (2)	Ni1—N1—C5—C13^i^	−44.1 (3)
N2^i^—Ni1—N1—C5	127.6 (2)	C7—N2—C6—N3	−0.9 (4)
N4—Ni1—N1—C5	−160.6 (2)	Ni1—N2—C6—N3	179.5 (2)
N4^i^—Ni1—N1—C5	35.10 (19)	C7—N2—C6—C4^i^	177.3 (3)
N1^i^—Ni1—N1—C5	−136.1 (2)	Ni1—N2—C6—C4^i^	−2.3 (4)
N2—Ni1—N1—C4	−172.7 (2)	C12—N3—C6—N2	0.9 (4)
N2^i^—Ni1—N1—C4	10.5 (2)	C12—N3—C6—C4^i^	−177.3 (3)
N4—Ni1—N1—C4	82.3 (3)	C6—N2—C7—C8	−179.7 (4)
N4^i^—Ni1—N1—C4	−82.0 (2)	Ni1—N2—C7—C8	−0.2 (6)
N1^i^—Ni1—N1—C4	106.8 (2)	C6—N2—C7—C12	0.5 (4)
N2—Ni1—N1—C3	64.4 (2)	Ni1—N2—C7—C12	180.0 (3)
N2^i^—Ni1—N1—C3	−112.4 (2)	C12—C7—C8—C9	1.3 (5)
N4—Ni1—N1—C3	−40.6 (3)	N2—C7—C8—C9	−178.5 (3)
N4^i^—Ni1—N1—C3	155.1 (2)	C7—C8—C9—C10	0.3 (6)
N1^i^—Ni1—N1—C3	−16.12 (14)	C8—C9—C10—C11	−1.3 (6)
N4—Ni1—N2—C6	74.0 (2)	C9—C10—C11—C12	0.8 (6)
N4^i^—Ni1—N2—C6	−164.6 (2)	C10—C11—C12—N3	178.2 (4)
N1^i^—Ni1—N2—C6	−4.9 (2)	C10—C11—C12—C7	0.8 (5)
N1—Ni1—N2—C6	−85.4 (2)	C6—N3—C12—C11	−178.2 (4)
N4—Ni1—N2—C7	−105.4 (3)	C6—N3—C12—C7	−0.5 (4)
N4^i^—Ni1—N2—C7	15.9 (3)	C8—C7—C12—C11	−1.9 (5)
N1^i^—Ni1—N2—C7	175.7 (4)	N2—C7—C12—C11	178.0 (3)
N1—Ni1—N2—C7	95.1 (3)	C8—C7—C12—N3	−179.8 (3)
N2—Ni1—N4—C13	−98.7 (2)	N2—C7—C12—N3	0.0 (4)
N2^i^—Ni1—N4—C13	77.1 (2)	C19—N4—C13—N5	0.4 (4)
N4^i^—Ni1—N4—C13	169.0 (2)	Ni1—N4—C13—N5	171.9 (2)
N1^i^—Ni1—N4—C13	−17.6 (2)	C19—N4—C13—C5^i^	−176.2 (3)
N1—Ni1—N4—C13	7.0 (4)	Ni1—N4—C13—C5^i^	−4.6 (4)
N2—Ni1—N4—C19	67.7 (3)	C14—N5—C13—N4	−0.7 (4)
N2^i^—Ni1—N4—C19	−116.5 (3)	C14—N5—C13—C5^i^	175.7 (3)
N4^i^—Ni1—N4—C19	−24.6 (3)	C13—N5—C14—C15	−175.8 (4)
N1^i^—Ni1—N4—C19	148.8 (3)	C13—N5—C14—C19	0.7 (4)
N1—Ni1—N4—C19	173.5 (3)	N5—C14—C15—C16	176.1 (4)
C1^i^—C1—C2—C3	57.5 (5)	C19—C14—C15—C16	0.1 (6)
C5—N1—C3—C2	−73.2 (4)	C14—C15—C16—C17	0.4 (6)
C4—N1—C3—C2	53.5 (4)	C15—C16—C17—C18	−0.8 (7)
Ni1—N1—C3—C2	173.5 (2)	C16—C17—C18—C19	0.7 (6)
C5—N1—C3—C3^i^	158.5 (3)	C17—C18—C19—N4	−176.0 (3)
C4—N1—C3—C3^i^	−74.8 (3)	C17—C18—C19—C14	−0.1 (5)
Ni1—N1—C3—C3^i^	45.2 (3)	C13—N4—C19—C18	176.4 (3)
C1—C2—C3—N1	−171.0 (3)	Ni1—N4—C19—C18	9.6 (6)
C1—C2—C3—C3^i^	−46.4 (5)	C13—N4—C19—C14	0.1 (3)
C5—N1—C4—C6^i^	−126.7 (3)	Ni1—N4—C19—C14	−166.7 (3)
C3—N1—C4—C6^i^	104.9 (3)	N5—C14—C19—C18	−177.2 (3)
Ni1—N1—C4—C6^i^	−13.8 (3)	C15—C14—C19—C18	−0.3 (5)
C4—N1—C5—C13^i^	72.2 (3)	N5—C14—C19—N4	−0.5 (4)
C3—N1—C5—C13^i^	−159.4 (3)	C15—C14—C19—N4	176.5 (3)

Symmetry codes: (i) −*x*, *y*, −*z*+3/2; (ii) −*x*, −*y*+1, −*z*+2.

### Hydrogen-bond geometry (Å, °)

**Table:**

*D*—H···*A*	*D*—H	H···*A*	*D*···*A*	*D*—H···*A*
N3—H3···O1^iii^	0.86	1.94	2.774 (10)	165
N5—H5···O2^iv^	0.86	2.14	2.923 (7)	151
C4—H4B···O4	0.97	2.37	3.268 (12)	154
C11—H11···Cg1^v^	0.93	2.73	3.542	147
C16—H16···Cg2^vi^	0.93	2.79	3.680	160

Symmetry codes: (iii) *x*−1, *y*, *z*; (iv) −*x*+1/2, −*y*+1/2, −*z*+1; (v) *x*+1/2, *y*+1/2, *z*+1; (vi) −*x*−1, −*y*−1, −*z*.
